# Endocrine-Disrupting Chemicals Exposure and Neurocognitive Function in the General Population: A Community-Based Study

**DOI:** 10.3390/toxics12070514

**Published:** 2024-07-17

**Authors:** Feng-Chieh Su, Yi-Chia Wei, Chiao-Yin Sun, Heng-Jung Hsu, Chin-Chan Lee, Yih-Ting Chen, Heng-Chih Pan, Cheng-Kai Hsu, Yun-An Liu, Chun-Yu Chen

**Affiliations:** 1 Department of Neurology, Chang Gung Memorial Hospital, Keelung 204, Taiwan; kukisu@cgmh.org.tw (F.-C.S.); 8905007@cgmh.org.tw (Y.-C.W.); 2 College of Medicine, Chang Gung University, Taoyuan 333, Taiwan; sun3970@cgmh.org.tw (C.-Y.S.); r5267@cgmh.org.tw (H.-J.H.); leefang@cgmh.org.tw (C.-C.L.); b9402031@cgmh.org.tw (Y.-T.C.); balour@cgmh.org.tw (H.-C.P.); kylegb@cgmh.org.tw (C.-K.H.); mp2522@cgmh.org.tw (Y.-A.L.); 3 School of Traditional Chinese Medicine, College of Medicine, Chang Gung University, Taoyuan 333, Taiwan; 4 Community Medicine Research Center, Chang Gung Memorial Hospital, Keelung 204, Taiwan; 5 Department of Nephrology, Chang Gung Memorial Hospital, Keelung 204, Taiwan

**Keywords:** endocrine-disrupting chemicals, phenol, phthalate, paraben, dementia, neurocognitive disorder

## Abstract

Background: Endocrine-disrupting chemicals (EDCs) are pervasive in everyday environments. The impacts of these chemicals, along with EDC-related lifestyle and dietary habits on neurocognitive function, are not well understood. Methods: The Chang Gung Community Medicine Research Center conducted a cross-sectional study involving 887 participants. From this initial cohort, 120 individuals were selected based on their EDC exposure scores for detailed analysis. Among these, 67 participants aged 55 years or older were further chosen to undergo cognitive impairment assessments using the Ascertain Dementia-8 (AD-8) questionnaire. Results: These 67 older participants did not significantly differ in age, albuminuria, or estimated glomerular filtration rate compared to those with lower impairment scores. This study revealed that mono-(2-ethylhexyl) phthalate (MEHP) levels (8.511 vs. 6.432 µg/g creatinine, *p* = 0.038) were associated with greater risk of cognitive impairment (AD-8 ≥ 2). Statistical models adjusting for age, gender, and diabetes indicated that MEHP levels positively correlated with AD-8 scores, achieving statistical significance in more comprehensive models (β ± SE: 0.160 ± 0.076, *p* = 0.042). Logistic regression analysis underscored a significant positive association between high MEHP levels and higher AD-8 scores (odds ratio: 1.217, *p* = 0.006). Receiver operating characteristic curves highlighted the association of high MEHP levels and EDC exposure scores for significant cognitive impairment, with areas under the curve of 66.3% and 66.6%, respectively. Conclusion: Exposure to EDCs, specifically di-(2-ethylhexyl) phthalate, the precursor to MEHP, may be associated with neurocognitive impairment in middle-aged and older adults.

## 1. Introduction

Endocrine-disrupting chemicals (EDCs) represent a class of compounds capable of interfering with the function of the endocrine system, as well as mimicking endogenous hormones. These substances may antagonize estrogen receptor interactions or disrupt the synthesis and metabolism of intrinsic female hormones [1]. EDCs are recognized as significant and expensive public health challenges due to their widespread presence and links to numerous chronic diseases. Most EDCs are lipophilic, accumulating in adipose tissue, which results in a prolonged half-life within the body. Additionally, a part of EDCs, also known as environmental estrogens, may originate from both natural and synthetic sources. Synthetic EDCs are particularly concerning due to their potential for more severe adverse health effects, highlighting their prioritization in recent public health policy initiatives aimed at mitigation [2].

Phthalates, a distinguished group within the category of endocrine-disrupting chemicals (EDCs), are prevalent plasticizers extensively used to improve the elasticity, pliability, and durability of polyvinyl chloride. Di-(2-ethylhexyl) phthalate (DEHP), a long-chain phthalate primarily utilized in PVC products, has the potential to seep into the environment gradually, thus becoming a common environmental contaminant [3,4]. In contrast, short-chain phthalates, such as dimethyl phthalate and diethyl phthalate, are often used in cosmetics as fragrance enhancers and in pharmaceuticals and personal care items [5]. The exposure to phthalates is associated with a variety of negative health effects, including infertility, premature thelarche, endometriosis, and asthma [6,7,8,9]. DEHP is known to interfere with the intrarenal renin-angiotensin system, potentially causing glomerulosclerosis, podocyte injury, and interstitial fibrosis [10]. Epidemiological research has established a connection between DEHP exposure and increased levels of microalbuminuria and β2-microglobulinuria, both indicators of renal impairment [11]. Moreover, preliminary findings from our ongoing community-based cohort study suggest that EDCs, including phthalates, parabens, and phenols, may contribute to the decline in renal function and exacerbate albuminuria over extended follow-up periods [12,13].

Gonadal hormones, including androgens and estrogens, are critical for cognitive function maintenance in adulthood and may reduce the risk of Alzheimer’s disease [14,15,16]. However, the impact of environmental EDCs that interfere with gonadal functions is frequently underestimated. EDCs appear as mimics of estrogens and androgens, as well as anti-estrogens and anti-androgens, among other forms. The brain, continually adapting throughout life to environmental changes and variations in gonadal steroid levels, is vulnerable to EDCs, which might hinder neurogenesis and jeopardize the maintenance of cognitive functions preservation in later life stages by disrupting the balance of gonadal hormones [15,17]. Furthermore, the brain is particularly susceptible to endocrine disruption during the gestational developmental period, which can lead to modified neuroendocrine hormone regulation, altered neurotransmitter system control, and significant behavioral changes, affecting learning, memory, and social interactions of the offspring [18]. Cohort studies have drawn connections between gestational EDC exposure and increased neurodevelopmental disorders rates. Organophosphate metabolites, phthalates, parabens, phenols, and per/polyfluoroalkyl substances are especially concerning, primarily because they disrupt thyroid hormone functions, potentially leading to growth retardation, cognitive deficits, attention-deficit hyperactivity disorder, and autism spectrum disorders [19,20,21,22].

Several studies have highlighted the disproportionately high exposure of the Taiwanese population to phthalate plasticizers compared to other nations [23,24]. In spite of this, investigations into the association between EDCs and neurocognitive function among elderly community dwellers are notably scarce. To bridge this research void, we initiated a community-based cohort study across four northeastern districts of Taiwan. This investigation included annual community visits to engage participants, comprising health assessments, blood and urine analyses, structured interviews concerning EDC exposure and neurocognitive disorder, and evaluations of urinary EDC concentrations. The primary objective of this study is to examine the potential connection between human EDC concentration and neurocognitive function.

## 2. Materials and Methods

### 2.1. Study Design 

Since November 2013, the Community Medicine Research Center (CMRC) has been conducting a prospective cohort study in the Gongliao, Ruifang, Wanli districts, and Keelung City of Northern Taiwan, which continues to this day. This study is designed to detect potential health issues at an early stage and address them effectively through consistent health evaluations, thereby providing a significant service to the community. Activities include blood collection, urinary tests, physical examinations, and surveys on health behaviors, alongside health seminars, hygiene education, community service activities, and interactive games.

### 2.2. Patient and Public Involvement

The outreach activities were conducted annually at health stations or community centers in various districts. Participants were recruited from among the community residents, who were then required to participate in the activities at intervals of 2–3 years. In 2019, we integrated an EDC survey into our program. Eligible participants were those aged over 18 years who participated in the health activities and were willing to undergo an EDC assessment and participate in a questionnaire interview. Informed consent was obtained from each participant before their inclusion in this study.

A team of trained interviewers administered a standardized questionnaire to all participants, gathering information on residential details, occupation, education, alcohol consumption, smoking habits, betel nut chewing tendencies, exercise habits, medical history, family history of diseases, and lifestyle practices that might increase the risk of EDC exposure and cognitive impairment. A total of 887 individuals participated in the EDC-focused questionnaire interview and underwent laboratory testing. From this group, 120 individuals were selected for further assessment of urinary EDC levels.

**Figure 1:**
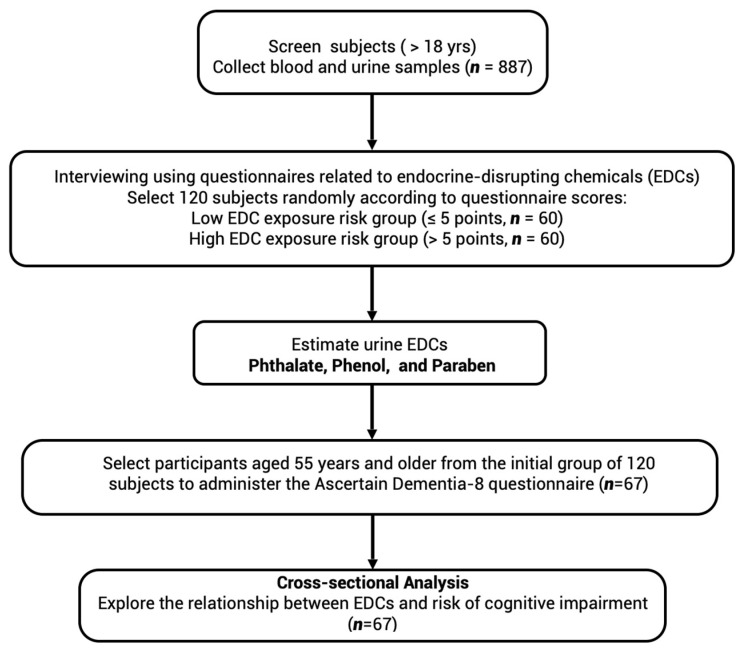
Flowchart of participants selected for urinary endocrine-disrupting chemicals estimation.

The cohort selected for further EDC analysis comprised 60 individuals randomly chosen from those categorized at low exposure risk (with questionnaire scores ≤ 5 points) and another 60 from the high exposure risk category (with questionnaire scores > 5 points). Subsequently, 67 participants aged 55 years or older were chosen for a post hoc analysis of cognitive impairment using the Ascertain Dementia–8 (AD-8) questionnaire (Figure 1).

Participant recruitment and sample preservation were integral components of the Northeastern Taiwan Community Medicine Research Cohort (NTCMRC, ClinicalTrials.gov Identifier: NCT04839796). This study was conducted in strict compliance with the Declaration of Helsinki and received approval from the Ethics Committee of the Institutional Review Board at Chang Gung Memorial Hospital (IRB: 201800275B0C602 and 201800289A3).

### 2.3. Questionnaires Concerning EDC Exposure and Potential Cognitive Impairment

The CMRC developed seven questionnaires to collect comprehensive data on the participants’ exposure to EDCs (Table S1). Additionally, the AD-8 questionnaire, crafted by the Alzheimer’s Disease Research Center at Washington University, includes eight items aimed at screening for dementing illnesses (Table S2). The AD-8 screening instrument consists of eight questions. Each “Yes, A change” response scores 1 point, while “NO, No change” or “N/A, Don’t know” responses score 0 points. The final score is derived by summing the items marked “Yes, A change”. A score of 0–1 indicates normal cognitive function, whereas a score of 2 or higher suggests the probable presence of cognitive impairment. Ten interviewers received specialized training to effectively administer these questionnaires. Their responsibilities included gathering detailed information on the consumption of foods potentially laden with EDCs, understanding the lifestyle practices of the participants, and predominantly administering the AD-8 questionnaire to informants. When administered to an informant, the interviewers specifically asked the respondent to rate changes observed in the participants.

### 2.4. Collection and Processing of Serum and Urine Samples

Participants were instructed to fast overnight before providing blood samples, which were then promptly transported to the laboratory for analysis within four hours of collection. Biochemical assays and complete blood counts were conducted on these samples. Subsequently, a subset of the samples was centrifuged at 3000× *g* for 10 min at a temperature of 4 °C in refrigerated tubes to separate the serum. This serum underwent further analysis, and samples exhibiting signs of lipemia or hemolysis were excluded from this study. The remaining serum samples were aliquoted and stored at −80 °C for subsequent analyses. Morning urine samples were also collected from the participants utilizing the midstream clean-catch technique.

### 2.5. Analysis of EDCs

The urinary samples were subjected to a detailed analysis for the detection of various EDCs, including phenols, parabens, phthalates, and benzophenone-3 (BP-3). The general molecular structures of these compounds are presented in Figure S1. The analysis targeted the following EDCs or their metabolites: (1) Phenols, such as bisphenol A (BPA), nonylphenol (NP), 4-tert-octylphenol (4-t-OP), and 2,4-di-tert-butylphenol (2,4-di-t-BP), along with triclosan and triclocarban; (2) Parabens, including methylparaben (MP), ethylparaben (EP), propylparaben (PP), and butylparaben (BP); (3) Phthalate metabolites, namely monomethyl phthalate (MMP), monoethyl phthalate (MEP), mono-(n-butyl) phthalate (MnBP), monobenzyl phthalate (MBzP), mono-(2-ethylhexyl) phthalate (MEHP), and mono-iso-nonyl phthalate (MiNP); (4) Other compounds, such as BP-3.

The sample preparation method was adapted from a protocol outlined by van der Meer et al., 2019 [25]. Initially, urine samples were thawed over a period of 24 h at a temperature of 4 °C. For the extraction of target compounds, a process was initiated by mixing a 100 μL aliquot of urine with 20 μL of methanol (MeOH) containing five stable isotope-labeled internal standards (D_7_-PP, ^13^C_4_-MEHP, ^13^C_4_-MiNP, ^13^C_12_-BPA, and ^13^C_6_-4-t-OP), 5 μL of β-glucuronidase (85,000 U/mL), and 20 μL of 1 M aqueous ammonium acetate, within a 1.5 mL microcentrifuge tube. This mixture was then vortexed for 10 s using a Vortex-2 Genie shaker (Scientific Industries, USA) to ensure a homogenous mixture. Following this, the mixture was incubated at 40 °C for one hour. To aid in the extraction process, 135 μL of 0.1% aqueous formic acid was introduced into the mixture, which was then processed through a supported liquid extraction cartridge. After the elution with 2.0 mL of dichloromethane, the extract was dried and subsequently reconstituted in a 200 μL solution of MeOH and H_2_O in equal parts. The prepared sample was finally analyzed using UPLC-MS/MS.

The quantification of 17 specific EDCs in human urine samples was accomplished using a Waters ACQUITY ultra-performance liquid chromatography (UPLC) system coupled with a SCIEX API-4000 triple quadrupole mass spectrometer, which featured an electrospray ionization source. These target compounds were identified and quantified employing multiple reaction monitoring (MRM) mode. The detection of BP-3 was carried out in the positive ionization mode, whereas the other 16 analytes were analyzed using negative ionization. Chromatographic separation of the target analytes was achieved on a Thermo Scientific Syncronis C^18^ column (150 × 2.1 mm, 3 μm). The UPLC mobile phases included 0.1% formic acid in Milli-Q water (for mobile phase A1, MPA-1), Milli-Q water alone (for mobile phase A2, MPA-2), and acetonitrile (for mobile phase B1, MPB-1). The analysis protocol for six phthalate metabolites, four parabens, triclosan, and triclocarban involved the use of MPA-1 and MPB-1 to elute 12 of the target analytes, while MPA-2 and MPB-1 facilitated the separation and ionization of BPA, BP-3, and three alkyl phenolic compounds. The column and sample tray temperatures were regulated at 30 °C and 4 °C, respectively, and the injection volume was fixed at 10 μL. The acquisition of data was managed through Analyst 1.6.2 software (SCIEX Corporation, Framingham, MA, USA)

To correct for urinary dilution, urinary analyte concentrations were adjusted by dividing the analyte concentration by the concentration of urinary creatinine. This standard approach helps account for variations in urine concentration among different samples. The quantification of urinary creatinine was performed using the Modified Jaffe, Kinetic method. This method involves a colorimetric assay where creatinine reacts with picric acid under alkaline conditions to form a yellow-orange complex. The reaction is monitored bichromatically at wavelengths of 520 nm and 800 nm, with the rate of change in absorbance being proportional to the creatinine concentration. The measurements were carried out using the Beckman AU5820 analyzer, ensuring the accurate assessment of creatinine levels for subsequent adjustment of analyte concentrations.

### 2.6. Validation of the Method for Measuring EDCs

This investigation aimed to assess the analytical characteristics of the UPLC-MS/MS technique, focusing on linearity, quantification limits (LOQs), and other metrics critical for ensuring quality assurance and control in the detection of 17 specific analytes in urine samples. To achieve reliable quantification, a matrix-matched calibration strategy employing six levels was adopted, utilizing synthetic urine as the blank matrix. The linearity of this method was established by the coefficient of determination (R2), with stable isotope-labeled internal standards (SIL-ISTDs) employed to calibrate retention times and correct for instrumental inaccuracies. The linear dynamic range for the target analytes varied: 10 to 1500 ng/mL for MEP, MP, triclosan, triclocarban, and benzophenone-3; 0.10 to 50.00 ng/mL for 4-t-OP and NP; and 1 to 500 ng/mL for the remaining ten analytes. The matrix-matched calibration curves for all 17 analytes followed a weighted (1/x) linear regression model, achieving an R2 greater than 0.990. The LOQs, with a signal-to-noise ratio (S/N) of at least 10, were established at 0.10 ng/mL for NP and 4-t-OP, with the remaining analytes having LOQs of 0.3 ng/mL. Precision and accuracy for intra- and inter-day analyses were evaluated by analyzing standard solutions five times within one day and across four consecutive days, respectively. The results for both intra- and inter-day precision and accuracy across all 17 analytes in synthetic urine met the guidelines recommended by the European Medicines Agency and the European Union.

### 2.7. Outcomes

This investigation sought to examine the association between urinary concentrations of EDCs and the risk of major neurocognitive disorder (MND) in individuals aged 55 or above, utilizing the AD-8 questionnaire as a tool for MND screening. The analysis integrated renal function assessments, albuminuria levels, demographic details, clinical characteristics, and EDC exposure information in conjunction with AD-8 screening outcomes.

### 2.8. Statistical Analysis

The normality of continuous variables was assessed through the Kolmogorov–Smirnov test, alongside measures of skewness and kurtosis. We compared demographics and clinical characteristics between groups with AD-8 scores ≥ 2 and <2 employing Student’s *t*-test for data following a normal distribution, and the Mann–Whitney U test for data not normally distributed. Categorical variables underwent evaluation via the chi-square test.

To investigate the associations between AD-8 scores and concentrations of endocrine-disrupting chemicals (EDCs), we implemented multiple linear regression analysis, adjusting for age, gender, diabetes, and renal function. Odds ratios for forecasting AD-8 scores ≥ 2 were computed using binary logistic regression, with adjustments for age and gender in Model 1, and a comprehensive adjustment for all variables in Model 2.

The utility of receiver operating characteristic (ROC) curves was employed to ascertain the connection of MEHP, MBzP, hemoglobin, or EDC exposure scores concerning AD-8 ≥ 2 occurrences. The area under the ROC curve (AUC) facilitated the evaluation of discriminative ability. Cut-off values for these variables were determined via ROC curves employing Youden’s index.

The association between AD-8 scores and EDC concentrations was further explored using correlation coefficients. All statistical analyses were bilateral, with a *p*-value of <0.05 deemed indicative of significance. Data processing utilized SPSS software version 27.0 for Mac, while GraphPad Prism version 10, SPSS, and Stata/MP 15.1 for Mac were employed for graph generation. 

## 3. Results

### 3.1. Study Characteristics

Among these 67 participants, those with AD-8 scores ≥ 2 demonstrated similar age, albuminuria, and estimated glomerular filtration rate (eGFR) (63.89 ± 5.77 vs. 64.83 ± 5.994 years old, *p* = 0.866; 6.80 (3.55–18.90) vs. 7.95 (4.73–23.78) mg/g, *p* = 0.526; 86.02 ± 16.19 vs. 84.81 ± 17.83 mL/min, *p* = 0.794), as well as comparable prevalence of diabetes, chronic kidney disease (15.8% vs. 25%, *p* = 0.415; 26.3% vs. 22.9%, *p* = 0.769), and levels of folate and vitamin B12 (11.58 ± 5.59 vs. 13.62 ± 8.47, *p* = 0.116; 684.86 ± 408.42 vs. 753.83 ± 536.09, *p* = 0.532) to the group with AD-8 scores < 2 (Table 1).

### 3.2. The Correlation between EDCs and AD-8 Assessment

**Figure 2:**
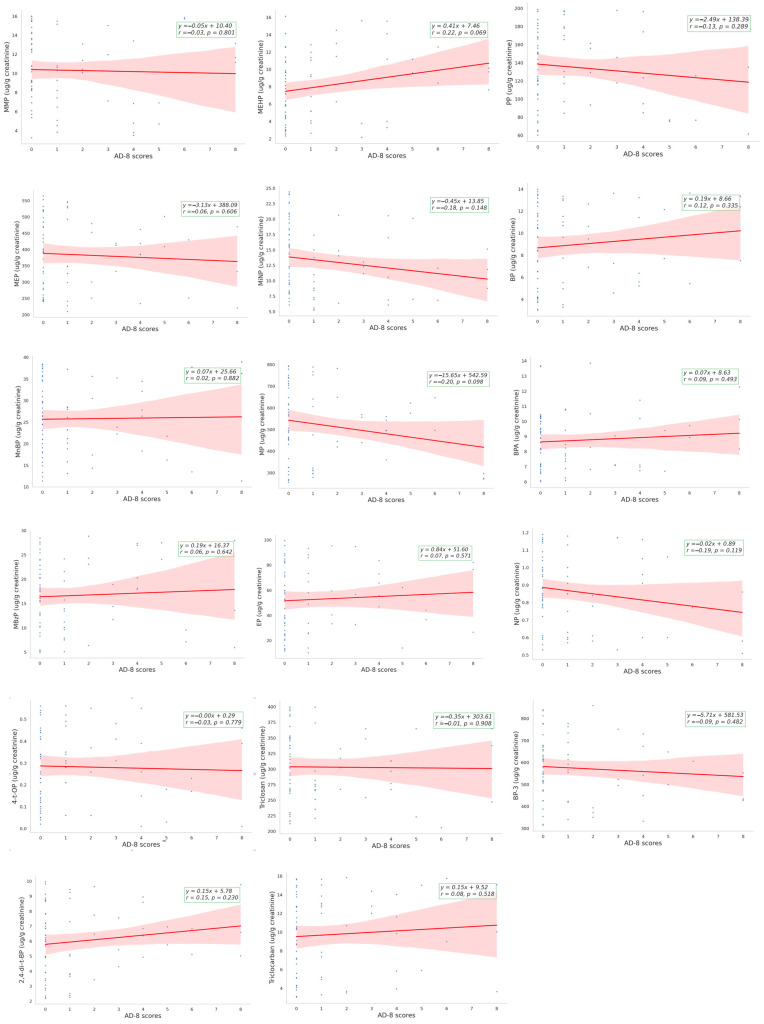
Associations between urinary endocrine-disruption chemicals levels and Ascertain Dementia-8 scores. The blue dots represent individuals’ AD-8 scores versus the corresponding endocrine-disrupting chemical concentrations. The red line represents the regression line of the blue dots, and the red area represents the 95% confidence interval of the regression line. Abbreviations: BPA, bisphenol A; NP, nonylphenol; 4-t-OP, 4-tert-octylphenol; 2,4-di-t-BP, 2,4-di-tert-butylpheno; MP, methylparaben; EP, ethylparaben; PP, propylparaben; BP, butylparaben; MMP, monomethyl phthalate; MEP, monoethyl phthalate; MnBP, mono-(n-butyl) phthalate; MBzP, monobenzyl phthalate; MEHP, mono-(2-ethylhexyl) phthalate; MiNP, monoisononyl phthalate; BP-3, benzophenone-3.

In this study, individuals in the AD-8 score ≥ 2 group exhibited higher levels of MEHP than those in the AD-8 < 2 group [8.511, 95% CI (6.436–11.26) vs. 6.432, 95% CI (5.485–7.544) µg/g creatinine, *p* = 0.038] (Table 2). Pearson correlation coefficients (*r*) detailing the relationship between 17 EDCs and AD-8 scores are presented in Figure 2. The correlations between MEHP, EP, NP, and BP-3 with AD-8 scores were 0.22 (*p* = 0.069), 0.07 (*p* = 0.571), −0.02 (*p* = 0.119), and −0.09 (*p* = 0.482), respectively. Although the small number of participants and the lack of adjustment for confounding factors make it difficult to present a clear linear relationship, MEHP, the primary compound of concern, still exhibits a tendency towards correlation, even though it is not statistically significant. Nevertheless, the above-mentioned four chemicals are known to associate with albuminuria and kidney function decline, as established in our prior cross-sectional study [13]. Multiple linear regression analysis further revealed that higher MEHP levels were positively associated with AD-8 scores in model 1 (β ± SE: 0.133 ± 0.067, *p* = 0.053, adjusted for age and gender) and reached statistical significance in model 2 (β ± SE: 0.160 ± 0.076, *p* = 0.042, adjusted for age, gender, diabetes, and the presence of 17 EDCs) (Table 3).

### 3.3. EDCs Level, EDC Exposure and Risk of Cognitive Impairment

Through binary logistic regression analyses of seven EDCs—specifically MEHP, MBzP, NP, BPA, MP, EP, and BP-3, which represent phthalates, parabens, and phenols—a combination of several clinically important characteristics was examined (Table 4). In Model 1, adjusted for age and gender, MEHP demonstrated a positive association with AD-8 scores ≥ 2 (OR: 1.168, 95% CI: 1.011–1.349), while hemoglobin showed a negative association (OR: 0.526, 95% CI: 0.289–0.957). In Model 2, which adjusted for all variables, a higher MEHP level was independently associated with AD-8 scores ≥ 2 (OR: 1.217, 95% CI: 1.011–2.421, *p* = 0.006).

**Figure 3:**
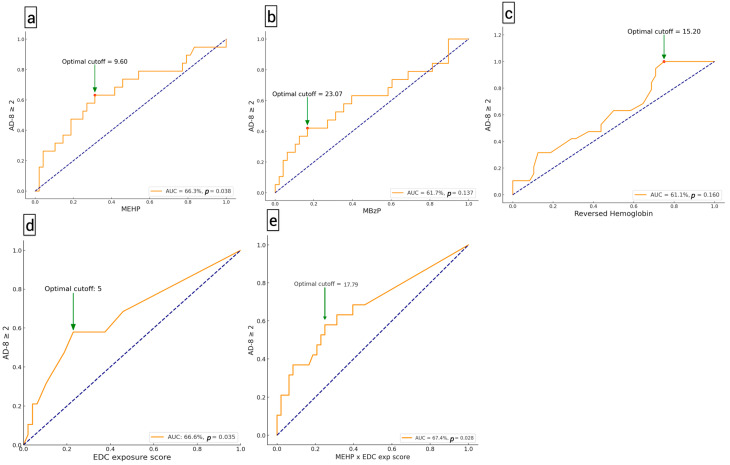
Receiver operating characteristic curves illustrating the performance of endocrine-disrupting chemicals (EDCs) to Ascertain Dementia-8 (AD-8) are presented. These include mono-(2-ethylhexyl) phthalate (MEHP) in (**a**), monobenzyl phthalate (MBzP) in (**b**), reversed hemoglobin in (**c**), EDC exposure score in (**d**), and the combined effect of MEHP and EDC exposure score in (**e**), obtained by multiplying both variables. These analyses focus on the development of Ascertain AD-8 scores of 2 or above, which indicate potential neurocognitive impairment. The blue dash lines represent diagonal reference lines.

The ROC curves, depicted in Figure 3, illustrate the association of high MEHP levels (AUC: 66.3%, *p* = 0.038), high EDC exposure scores (AUC: 66.6%, *p* = 0.035), and their combination (AUC: 67.4%, *p* = 0.028) for AD-8 scores ≥ 2. These results suggest that MEHP levels higher than the cutoff value of 9.6 µg/g creatinine and EDC exposure scores higher than the cutoff value of 5 points are significantly associated with impaired cognitive function. These findings also indicate a potential interaction between MEHP levels and lifestyle or dietary patterns related to EDC exposure, which may influence the occurrence of cognitive impairment. However, higher MBzP and lower hemoglobin also tended to be associated with AD-8 scores ≥ 2, although they did not show significance in the ROC curve analysis.

Moreover, the AD-8 ≥ 2 group displayed higher EDC exposure scores compared to the AD-8 < 2 group (5.21 ± 5.49 vs. 2.33 ± 3.77, *p* = 0.060). Within the subgroup analysis, individuals with EDC exposure scores greater than 5 showed a significantly higher probability of having AD-8 scores ≥ 2 compared to those with scores of 5 or less (47.4% vs. 18.8%, *p* = 0.017) (Table 5).

## 4. Discussion

This study marks the first examination of EDCs combined with cognitive impairment screening in a community-based cohort in Taiwan’s general population. A key finding from our research is that high concentrations of MEHP, a metabolite of DEHP, were significantly positively associated with AD-8 scores ≥ 2 completed by the informant, indicative of potential cognitive impairment. Additionally, EDC exposure scores, derived from questionnaire interviews, also showed a positive correlation with AD-8 scores. Overall, the results suggest that exposure to EDCs, particularly DEHP, may adversely affect neurocognitive functions, as evidenced by this cross-sectional study.

The AD-8 is a concise screening instrument designed to detect mild cognitive impairment (MCI) and MND. It utilizes the Clinical Dementia Rating (CDR), a gold-standard informant-based scale that is extensively employed in research settings to diagnose and stage the severity of neurocognitive impairment [26,27]. Particularly beneficial for elderly adults in community settings, AD-8 screening enhances our understanding of mental health and aids in the early detection and treatment of cognitive impairments in the general population. The AD-8 has demonstrated high sensitivity (90.9%) and specificity (89.0%) for MND in community research settings, although its association with MCI is somewhat limited, with an AUC of 64.5% [28]. Conversely, a study from Turkey highlighted that AD-8 could effectively screen for both MCI (AUC: 97.9%) and MND (AUC: 99.9%), confirmed by the CDR scale and the Mini-Mental State Examination (MMSE) [29]. A systematic review also revealed that informant-completed AD-8 is superior to participant-completed forms, showing a sensitivity of 80% and specificity of 79% in detecting MCI [30]. Therefore, AD-8 is a quick, simple, and sensitive method for detecting both minor and major cognitive impairments, and it does not require highly trained professionals to administer more complex tools like the CDR and MMSE, making it more feasible for large-scale cognitive screening in community settings.

The full impact of human exposure to EDCs is difficult to assess because adverse effects often develop latently and only become apparent at later ages, and in some cases, may not present at all [2]. EDCs are known to disrupt gonadal hormones, exert obesogenic effects, and interfere with both the hypothalamic–pituitary–thyroid and hypothalamic–pituitary–adrenal (HPA) axes [31,32,33,34,35]. Neurogenesis during developmental periods is significantly regulated by thyroid hormones, and dysregulation of the HPA axis is strongly associated with major depressive disorder, characterized by impaired immunomodulatory effects of glucocorticoids, which may lead to neuroinflammation [36,37]. Consequently, EDCs are considered to pose neurotoxic risks.

Our study has unveiled a significant positive association between urinary MEHP levels and AD-8 scores, utilizing both simple two-group tests (AD-8 ≥2 vs. <2) and multiple linear regression that adjusts for age, gender, and renal function. Additionally, the EDC exposure score also shows a trend of positive correlation with AD-8 scores, suggesting that lifestyle and dietary habits related to EDC exposure might reflect the extent of cognitive impairment. Multivariable logistic regression and ROC curve analyses have both suggested that high MEHP levels, defined by a cutoff value of over 9.6 µg/g creatinine obtained from ROC curve analyses, significantly correlate with AD-8 scores ≥ 2. This metric helps quantify the accuracy of MEHP as a potential predictor for cognitive impairment in our study population. Furthermore, ROC curve analysis has determined a cutoff value of 5 for EDC exposure scores, which indicate the occurrence of AD-8 scores ≥ 2, suggesting a high risk for impaired cognitive function in middle-aged and older adults with high urinary MEHP concentrations and exposure scores. In addition, the combination of MEHP levels and EDC exposure scores yielded an AUC of 67.4%, reinforcing the notion of their joint influence on cognitive outcomes. While these results highlight MEHP’s significance, they also imply that broader EDC exposure patterns, encapsulated by the exposure scores, may amplify this association. These findings illuminate that exposure to EDCs, particularly DEHP, may be linked to cognitive impairment. This may be attributed to the disruption of gonadal hormones by EDCs, which are critical in regulating the aging brain and pivotal to the processes of neurogenesis and the preservation of neurocognitive function in later life [17]. To our knowledge, our study is the first to explore the exposure of community residents to EDCs in combination with a cognitive function questionnaire and an EDC exposure interview, yielding results that align with previous laboratory and animal studies.

Early-life exposure to EDCs is associated with significant societal impacts due to neurobehavioral consequences. However, the influence of EDCs is notably profound in later life, driven by two potential mechanisms. First, early exposure may induce “silent damage” that remains clinically undetected until aging diminishes the brain’s compensatory abilities, at which point functional impairments emerge [17]. Second, EDC exposure during advanced age may worsen the neurobehavioral impacts of declining hormonal production, a natural aspect of aging. Moreover, gonadal hormones are crucial for maintaining optimal brain function throughout adulthood and into senescence [38]. Decreases in androgen levels are well documented as a risk factor for cognitive decline and Alzheimer’s disease [39,40,41]. Several studies in animal models demonstrate that gonadal hormones provide protective effects against neurodegenerative disorders such as dementia and support cognitive function [42,43,44]. Conversely, the anti-androgenic or anti-estrogenic properties of EDCs could heighten this vulnerability. The HPA axis is also vulnerable to disruption by EDCs. Adrenal corticosteroids, which are vital for neurogenesis, find the adrenal gland particularly susceptible due to its high vascularization, significant lipophilicity due to fatty acid content, and the presence of cytochrome P450 enzymes that generate free radicals and toxic compounds [45]. The human adrenocortical cell line H295R, which expresses all enzymes necessary for steroidogenesis, serves as a prevalent model for EDC assays [46]. EDCs can cause adrenocortical toxicity by either stimulating or inhibiting steroidogenic enzymes. Although the precise mechanisms by which EDCs impair neurocognitive function are not fully understood, the disruption of gonadal and adrenocortical hormone balance likely plays a crucial role.

We used the traditional approach of dividing analyte concentration by creatinine to address urinary dilution, which may produce some biased results, especially in heterogeneous populations. However, our study population mostly comprised residents from nearby communities who were relatively healthy, mobile, and of similar age, which likely reduced individual heterogeneity to some extent. Alternative methods, such as using unadjusted analyte concentrations in statistical models, adjusting for creatinine as a covariate, or using covariate-adjusted standardization, may provide more accurate results [47]. Future studies should consider these methods to minimize potential biases.

The current study has several limitations. First, this cross-sectional study inherently faces the limitation of potential reverse causality. Given the relatively short half-lives of EDCs in the body, establishing a direct causative link to long-term neurocognitive outcomes is challenging. We hypothesize that continuous or repeated exposure to EDCs, reflected in lifestyle and dietary habits, may contribute to cumulative effects over time, potentially impacting cognitive function. Second, our assessment was limited to the AD-8 questionnaire screen, and we did not employ additional screening tools like the CDR or the MMSE conducted by a clinical psychologist or a formal neuropsychological test by a neurologist to ascertain alignment with the Diagnostic and Statistical Manual of Mental Disorders, Fifth Edition (DSM-5) criteria for MND. However, the AD-8 screen does offer approximately 90% sensitivity and specificity for detecting MND, and complete laboratory tests including vitamin B12, folate, and complete blood cell count were performed, which to some extent may exclude cognitive impairments associated with these conditions. Thus, we believe that the AD-8 still provides valuable early detection of cognitive disorders within the community. For residents with potential prodromal cognitive impairment or MND scoring AD-8 ≥ 2, referrals to medical institutions for definitive diagnosis are still pursued. Third, the results of urinary EDC measurements may have been influenced by the participants’ dietary and lifestyle habits in the days leading up to sample collection. The urine samples were derived from spot urine, not 24 h collections, introducing potential bias. However, our EDC exposure questionnaire design, based on frequency, suggests that the participants’ lifestyle and dietary habits remained comparable. Additionally, the association between EDC exposure and questionnaire scores, established in our previous cross-sectional analysis, and the combined analysis of the EDC exposure questionnaire with the AD-8 questionnaire in this study, could potentially counterbalance the limitation of urine sample timing [12]. Fourth, a relatively small subset of participants was selected for urinary EDC measurement and the AD-8 screen, which may not ideally represent the linear relationship and normal distribution of exposure. This subset focused on relatively healthy residents aged 55 or older, with the aim of identifying very early stages of cognitive impairment.

## 5. Conclusions

This cross-sectional observational study conducted by the CMRC substantiates the influence of EDCs on neurocognitive function. Our findings reveal a significant correlation between higher urinary levels of EDCs, especially DEHP, and an elevated risk of cognitive impairment.

Notably, MEHP emerged as a pivotal determinant, demonstrating a robust linear association with total AD-8 scores. This indicates that the severity of neurocognitive disorder is positively correlated with urinary MEHP concentration, independent of age, gender, diabetes, and renal function. Participants with AD-8 scores ≥ 2 also showed a higher proportion of EDC exposure scores > 5. This suggests that lifestyle and dietary habits related to EDC exposure may confer a higher risk of cognitive impairment.

These findings underscore the necessity for further research into the relationship between environmental EDC exposure and community mental health. Given the widespread distribution of EDCs, their potential impact on the neurocognitive function of the aging brain deserves significant public health consideration. This study thereby reinforces the legitimacy and necessity of proactive early MCI screening in the community and preemptive strategies in populations highly exposed to EDCs, to mitigate the decline of cognitive function.

## Figures and Tables

**Table 1:** Demographics and clinical characteristics of study population testing the urinary EDCs.

	AD-8 Score ≥ 2 (*n* = 19)	AD-8 Score < 2 (*n* = 48)	*p* Value
Age (years)	63.89 ± 5.772	64.83 ± 5.994	0.866
Male, *n*, (%)	5 (26.3)	17 (35.4)	0.475
Diabetes, *n*, (%)	3 (15.8)	12 (25)	0.415
Dyslipidemia, *n*, (%)	16 (84.2)	42 (87.5)	0.722
CKD, *n*, (%)	5 (26.3)	11 (22.9)	0.769
Hypertension, *n*, (%)	13 (68.4)	38 (79.2)	0.352
White blood cell (1000/μL)	5.38 ± 0.92	6.00 ± 1.77	0.029 *
Hemoglobin(g/dL)	13.86 ± 1.07	14.45 ± 1.18	0.595
Ferritin (ng/mL)	254.47 ± 155.54	246.93 ± 203.33	0.682
Folate (ng/mL)	11.59 ± 5.59	13.62 ± 8.47	0.116
AST (U/L)	24.05 ± 6.74	23.04 ± 5.83	0.964
ALT (U/L)	30.26 ± 18.32	28.13 ± 12.64	0.531
Total bilirubin (mg/dL)	0.62 ± 0.21	0.68 ± 0.31	0.407
Insulin (uU/mL)	12.39 ± 8.44	12.39 ± 7.5	0.507
Cholesterol (mg/dL)	197.11 ± 29.814	199.33 ± 35.19	0.615
HS-CRP (mg/L)	1.38 ± 1.18	1.94 ± 2.98	0.518
Bun (mg/dL)	14.78 ± 4.79	15.56 ± 5.95	0.603
Creatinine (mg/dL)	0.77 ± 0.20	0.81 ± 0.26	0.628
eGFR (mL/min/1.73 m^2^)	86.02 ± 16.19	84.81 ± 17.83	0.794
Na (mEq/L)	142.63 ± 1.50	141.88 ± 1.53	0.954
K (mEq/L)	4.48 ± 0.35	4.49 ± 0.33	0.908
Ca (mg/dL)	9.55 ± 0.31	9.51 ± 0.26	0.267
P (mg/dL)	3.71 ± 0.54	3.60 ± 0.54	0.683
Albumin (g/dL)	4.69 ± 0.29	4.69 ± 0.24	0.533
Vitamin D (ng/mL)	28.06 ± 7.76	31.08 ± 9.53	0.846
Vitamin B12 (pg/mL)	684.86 ± 408.42	753.83 ± 536.09	0.532
Folate (ng/mL)	11.58 ± 5.59	13.62 ± 8.47	0.116
Body-mass index	25.76 ± 5.12	26.21 ± 3.87	0.406
UACR (mg/g)	6.80 (3.55–18.90)	7.95 (4.73–23.78)	0.526
UPCR (mg/g)	63.27 (50.42–78.17)	72.55 (59.03–93.19)	0.117
HOMA-IR	3.73 ± 4.30	3.52 ± 2.91	0.343
Leptin (ng/mL)	9.25 ± 6.18	11.49 ± 6.90	0.740

Abbreviations: AD-8, Ascertain Dementia-8 questionnaire; EDCs, endocrine-disrupting chemicals; CKD, chronic kidney disease; AST, aspartate transaminase; ALT, alanine transaminase; HS-CRP, high sensitivity C-reactive protein; eGFR, estimated glomerular filtration rate; UACR, urine albumin-to-creatinine ratio; UPCR, urine protein-to-creatinine ratio; HOMA-IR, homeostasis model assessment-insulin resistance index. *: *p* value < 0.05.

**Table 2:** Urinary EDCs of study population.

	AD-8 Score ≥ 2 (*n* = 19)	AD-8 Score < 2 (*n* = 48)	*p* Value
Urinary XEs (ug/g creatinine)			
BPA	8.759 (7.873–9.745)	8.473 (7.995–8.980)	0.646
NP	0.787 (0.686–0.902)	0.857 (0.802–0.915)	0.284
4-t-OP	0.179 (0.097–0.327)	0.223 (0.176–0.282)	0.856
2,4-di–t-BP	6.411 (5.607–7.331)	5.214 (4.555–5.968)	0.160
Triclosan	299.7 (275.5–326.0)	297.8 (282.3–314.2)	0.878
Triclocarban	8.952 (6.866–11.67)	8.601 (7.443–9.939)	0.559
MP	480.5 (416.2–554.7)	496.3 (445.7–552.6)	0.487
EP	51.46 (40.61–65.21)	42.98 (35.65–51.83)	0.396
PP	119.6 (100.5–142.3)	131.4 (119.4–144.6)	0.381
BP	8.837 (7.366–10.60)	7.871 (6.828–9.074)	0.436
MMP	9.09 (7.191–11.49)	9.746 (8.687–10.93)	0.760
MEP	365.0 (321.9–413.9)	369.7 (338.2–404.2)	0.666
MnBP	24.33 (20.18–29.33)	24.37 (22.12–26.86)	0.900
MBzP	16.64 (12.93–21.43)	14.27 (12.38–16.46)	0.137
MEHP	8.511 (6.436–11.26)	6.432 (5.485–7.544)	0.038 *
MiNP	11.47 (9.437–13.95)	12.32 (10.80–14.05)	0.461
BP-3	528.1 (460.4–605.7)	566.3 (526.4–609.3)	0.323

Notes: Data are presented as geometric mean (95% confidence interval). Abbreviations: Ascertain Dementia-8 questionnaire, AD-8; EDCs, endocrine-disrupting chemicals; BPA, bisphenol A; NP, nonylphenol; 4-t-OP, 4-tert-octylphenol; 2,4-di-t-BP, 2,4-di-tert-butylpheno; MP, methylparaben; EP, ethylparaben; PP, propylparaben; BP, butylparaben; MMP, monomethyl phthalate; MEP, monoethyl phthalate; MnBP, mono-(n-butyl) phthalate; MBzP, monobenzyl phthalate; MEHP, mono-(2-ethylhexyl) phthalate; MiNP, monoisononyl phthalate; BP-3, benzophenone-3. *: *p* value < 0.05.

**Table 3:** β-coefficient between AD-8 scores and independent variables (*n* = 67).

	Simple Linear Regression		Multiple Regression Analysis		Multiple Regression Analysis	
			Model 1		Model 2	
	β ± SE	*p*	β ± SE	*p*	β ± SE	*p*
Age	0.014 ± 0.046	0.769	—	—	0.006 ± 0.055	0.907
Gender	−0.555 ± 0.568	0.332	—	—	−0.231 ± 0.689	0.739
Diabetes	−0.710 ± 0.638	0.270	−0.692 ± 0.645	0.287	−1.244 ± 0.740	0.099
eGFR	0.001 ± 0.016	0.948	0.003 ± 0.016	0.876	0.011 ± 0.019	0.562
BPA	0.101 ± 0.146	0.491	0.088 ± 0.147	0.552	−0.029 ± 0.178	0.870
NP	−2.030 ± 1.311	0.126	−1.796 ± 1.386	0.200	−2.638 ± 1.612	0.109
4-t-OP	−0.427 ± 1.626	0.794	−0.336 ± 1.678	0.842	0.875 ± 2.135	0.684
2,4-di–t-BP	0.144 ± 0.119	0.230	0.151 ± 0.121	0.219	0.158 ± 0.143	0.273
Triclosan	−0.001 ± 0.005	0.908	−0.001 ± 0.005	0.897	−0.001 ± 0.006	0.856
Triclocarban	0.042 ± 0.065	0.518	0.031 ± 0.067	0.641	0.055 ± 0.075	0.463
MP	−0.003 ± 0.002	0.098	−0.002 ± 0.002	0.133	−0.005 ± 0.002	0.020 *
EP	0.006 ± 0.010	0.571	0.007 ± 0.011	0.502	0.015 ± 0.013	0.250
PP	−0.007 ± 0.006	0.289	−0.007 ± 0.007	0.274	−0.009 ± 0.008	0.228
BP	0.074 ± 0.076	0.335	0.083 ± 0.077	0.287	0.067 ± 0.092	0.471
MMP	−0.018 ± 0.073	0.801	−0.005 ± 0.079	0.945	−0.025 ± 0.094	0.794
MEP	−0.001 ± 0.003	0.606	−0.002 ± 0.003	0.539	−0.001 ± 0.003	0.703
MnBP	0.005 ± 0.033	0.882	0.007 ± 0.033	0.837	0.000 ± 0.038	0.993
MBzP	0.018 ± 0.038	0.642	0.022 ± 0.039	0.581	0.018 ± 0.043	0.677
MEHP	0.123 ± 0.067	0.069	0.133 ± 0.067	0.053	0.160 ± 0.076	0.042 *
MiNP	−0.071 ± 0.049	0.148	−0.061 ± 0.054	0.262	−0.026 ± 0.063	0.681
BP-3	−0.001 ± 0.002	0.482	−0.001 ± 0.002	0.561	0.002 ± 0.002	0.446

Abbreviations: eGFR, estimated glomerular filtration rate; BPA, bisphenol A; NP, nonylphenol; 4-t-OP, 4-tert-octylphenol; 2,4-di-t-BP, 2,4-di-tert-butylpheno; MP, methylparaben; EP, ethylparaben; PP, propylparaben; BP, butylparaben; MMP, monomethyl phthalate; MEP, monoethyl phthalate; MnBP, mono-(n-butyl) phthalate; MBzP, monobenzyl phthalate; MEHP, mono-(2-ethylhexyl) phthalate; MiNP, monoisononyl phthalate; BP-3, benzophenone-3. Model 1: adjusted for age and gender, Model 2: adjusted for age, gender, diabetes, eGFR, and 17 endocrine-disrupting chemicals. *: *p* value < 0.05.

**Table 4:** Univariate and multivariable analysis of the EDC in relation to AD-8 score ≥ 2 (*n* = 67).

	Univariate (*n* = 67)			Multivariable, Model 1			Multivariable, Model 2		
	Crude OR	95% CI	*p* Value	Adjusted OR	95% CI	*p* Value	Adjusted OR	95% CI	*p* Value
MEHP	1.168	1.011–1.349	0.035 *	1.175	1.014–1.362	0.032 *	1.217	1.011–1.464	0.038 *
MBzP	1.059	0.979–1.146	0.150	1.071	0.985–1.163	0.107	1.083	0.978–1.200	0.127
NP	0.222	0.015–3.290	0.274	0.220	0.013–3.596	0.288	0.025	0.001–1.214	0.063
BPA	1.100	0.828–1.462	0.512	1.095	0.821–1.460	0.538	0.949	0.665–1.353	0.771
MP	0.999	0.996–1.002	0.524	0.999	0.996–1.002	0.615	0.998	0.994–1.003	0.522
EP	1.009	0.988–1.030	0.422	1.009	0.988–1.031	0.386	1.025	0.994–1.057	0.111
BP-3	0.998	0.994–1.002	0.370	0.998	0.994–1.002	0.355	0.999	0.994–1.004	0.804
Age	0.972	0.884–1.068	0.556	—	—	—	0.907	0.786–1.046	0.179
Gender	0.651	0.200–2.120	0.476	—	—	—	1.087	0.195–6.056	0.924
eGFR	1.001	0.973–1.036	0.795	1.003	0.970–1.036	0.870	1.018	0.973–1.065	0.440
Diabetes	0.563	0.139–2.271	0.419	0.549	0.135–2.232	0.402	0.230	0.027–1.966	0.180
Vitamin D	0.959	0.898–1.026	0.224	0.962	0.899–1.029	0.261	0.993	0.908–1.086	0.880
Hemoglobin	0.626	0.378–1.037	0.069	0.526	0.289–0.957	0.036 *	0.521	0.237–1.145	0.104

Abbreviations: EDCs, endocrine-disrupting chemicals; AD-8, Ascertain Dementia-8 questionnaire; MEHP, mono-(2-ethylhexyl) phthalate; MBzP, monobenzyl phthalate; NP, nonylphenol; BPA, bisphenol A; MP, methylparaben; EP, ethylparaben; BP-3, benzophenone-3; eGFR, estimated glomerular filtration rate. Model 1: adjustment for age and gender, Model 2: adjustment for age, gender, and all variables listed in the first column of the table. *: *p* value < 0.05.

**Table 5:** Associations between EDC exposure scores and AD-8 questionnaires.

	AD-8 Scores ≥ 2 (*n* = 19)	AD-8 Score < 2 (*n* = 48)	*p* Value
EDC exp score > 5 (*n* = 18), *n* (%)	9 (47.4)	9 (18.8)	0.017 *
EDC exposure scores	5.21 ± 5.493	2.33 ± 3.772	0.060

Abbreviations: EDC exp, endocrine-disrupting chemicals exposure questionnaire; AD-8, Ascertain Dementia-8 questionnaire. *: *p* value < 0.05.

## Data Availability

Data are contained within the article and Supplementary Materials.

## Supplementary Materials

The following supporting information can be downloaded at: https://www.mdpi.com/article/10.3390/toxics12070514/s1, Table S1: Endocrine-Disrupting Chemicals Exposure Screen Interview, Table S2: Ascertain Dementia-8 (AD-8) Screening Interview, Figure S1: Panel a, general molecular structure of phthalates (R and R′ are general place holders.); Panel b, general molecular structure of parabens (a para-hydroxybenzoate) where R = an alkyl group; Panel c, General molecular structure of a phenols; Panel d, general molecular structure of benzophnone-3.
