# Supplementary material for: Endocrine-Disrupting Chemicals Exposure and Neurocognitive Function in the General Population: A Community-Based Study
Source: Toxics. 2024 Jul 17;12(7):514. doi: 10.3390/toxics12070514 (PMC11281080; doi:10.3390/toxics12070514)

**Table S1**

Endocrine-Disrupting Chemicals Exposure Screen Interview

| | less than one day per week *0 point* | 1-2 days per week *1 point* | 3-4 days per week *2 points* | 5-7 days per week *3 points* |
|---|---|---|---|---|
| How often do you use plastic bags for hot food? | | | | |
| How often do you use plastic tableware? | | | | |
| How often do you consume packaged processed foods? | | | | |
| Do you use plastic wrap for microwaving, steaming, or packaging oily food at home? | | | | |
| On how many days per week do you drink a beverage in a commercially available plastic cup? | | | | |
| Do you use fragranced bathroom products? | | | | |
| Do you use plastic floor mats in your home? | | | | |
| TOTAL SCORES | | | | |

The questionnaire was designed by Community Medicine Research Center, Chang Gung Memorial Hospital, Keelung Branch 2018

**Table S2**

Ascertain Dementia-8 (AD-8) Screening Interview

| Remember, "Yes, a change" indicates that there has been a change in the last several years caused by cognitive (thinking and memory) problems. | YES, A change (1 point) | NO, No change (0 point) | N/A, Don't know (0 point) |
|---|---|---|---|
| 1. Problems with judgment (e.g., problems making decisions, bad financial decisions, problems with [illegible] | | | |
| 2. Less interest in hobbies/activities | | | |
| 3. Repeats the same things over and over (questions, stories, or statements) | | | |
| 4. Trouble learning how to use a tool, appliance, or gadget (e.g., VCR, computer, microwave, remote control) | | | |
| 5. Forgets correct month or year | | | |
| 6. Trouble handling complicated financial affairs (e.g., balancing checkbook, income taxes, paying bills) | | | |
| 7. Trouble remembering appointments | | | |
| 8. Daily problems with thinking and/or memory | | | |
| TOTAL AD-8 SCORE | | | |

Adapted from Galvin JE et al, The AD-8, a brief informant interview to detect dementia, Neurology 2005:65:559-564

Copyright 2005. The AD8 is a copyrighted instrument of the Alzheimer's Disease Research Center, Washington University, St. Louis, Missouri. All Rights Reserved.

Figure S1. Panel a, general molecular structure of phthalates (R and R' are general place holders.); Panel b, general molecular structure of parabens (a para-hydroxybenzoate) where R = an alkyl group; Panel c, General molecular structure of a phenols; Panel d, general molecular structure of benzophnone-3

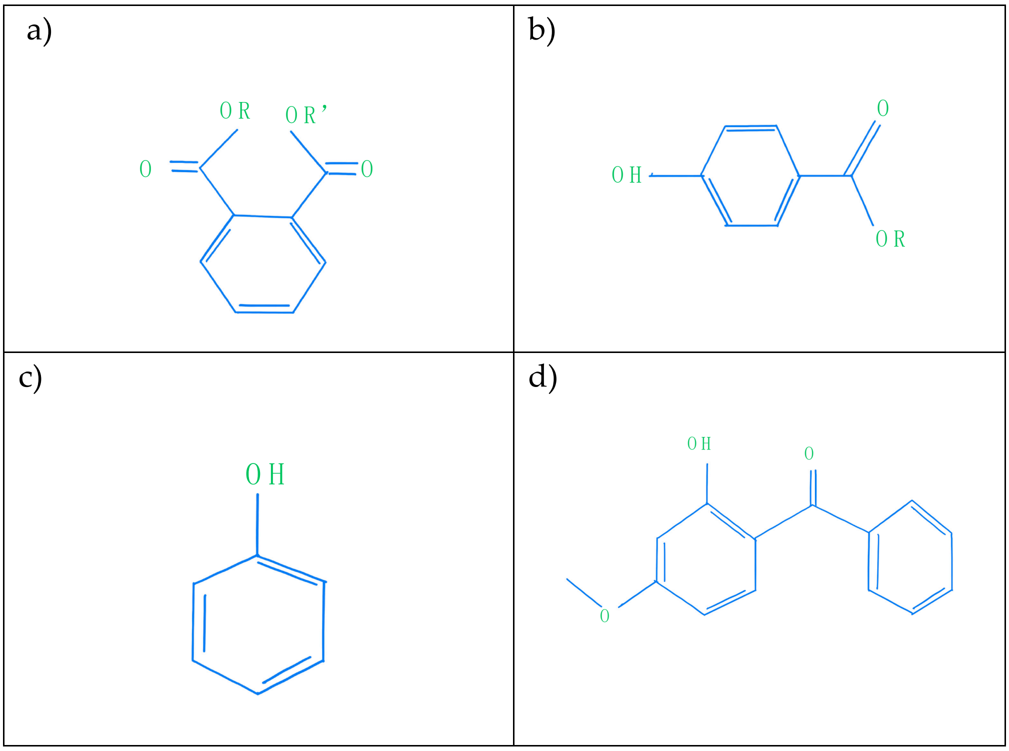

Supplement: Supplementary file 1 [file toxics-12-00514-s001.zip › toxics-3086979-supplementary.pdf]
